# In the pubertal rat, the regulation of ovarian function involves the synergic participation of the sensory and sympathetic innervations that arrive at the gonad

**DOI:** 10.1186/s12958-015-0062-8

**Published:** 2015-06-17

**Authors:** Leticia Morales-Ledesma, Angélica Trujillo, Javier Apolonio

**Affiliations:** Biology of Reproduction Research Unit, Physiology of Reproduction Laboratory, Facultad de Estudios Superiores Zaragoza, UNAM, AP 9–020, CP 15000 México, D. F, México; Benemérita Universidad Autónoma de Puebla, Escuela de Biología, Edificio 112A Ciudad Universitaria, CP 72570 Puebla, Puebla Mexico

**Keywords:** Steroid secretion, Puberty, Sensorial innervation, Sympathetic innervation, Ovary

## Abstract

**Background:**

The present study investigates sectioning the superior ovarian nerve (SON) in rats with functional sensorial denervation induced by capsaicin administration at birth and the effects on the establishment of puberty, ovulation, serum progesterone, and estradiol concentrations.

**Methods:**

The animals were allotted randomly to one of the following experimental groups. Groups of 8–10 rats were injected at birth with capsaicin or vehicle, and on day 20 or 28 of life, they were submitted to a sham operation (SO). Other groups of 8–10 rats were injected at birth with capsaicin or vehicle, and on day 20 or 28 of life, they were submitted to the uni-or bilateral SON sectioning. The animals were killed at the first estrus. Serum concentration of progesterone (ng/ml) and estradiol (pg/ml) were measured using a radioimmunoassay.

**Results:**

Animals treated with capsaicin and subjected at 20 days of life to the left or bilateral section of SON had a delayed age of vaginal opening. Furthermore, animals with a lack of sensory information and subjected to a SO at 28 days of life had the same delay in the age of vaginal opening. Animals with sensorial innervation intact, subjected to unilateral section of the SON at 20 or 28 days of age, showed diminished ovulation rate and number of ova shed by the denervated ovary. In animals with sensorial denervation, the uni-or bilateral sectioning of the SON did not result in changes in ovulation. Progesterone and estradiol levels were different depending on the age of the animal in which the SON section was performed.

**Conclusions:**

Based on the present results, we suggest that sympathetic innervation regulates ovulation and the secretion of steroid hormones and that the sensory fibers modulate the sympathetic innervation action on ovarian functions.

## Background

Ovarian functions are regulated by the central nervous system (CNS) through the release of the GnRH, which in turn regulates the secretion of luteinizing hormone (LH) and follicle-stimulating hormone (FSH) from the pituitary. LH and FSH stimulate the secretion of steroid hormones from the ovaries [1]. Also the noradrenergic ovarian innervation arriving through the superior ovarian nerve (SON) and the ovarian plexus nerve play a role in the regulation of ovarian functions [2]. Additionally, neural pathways between the ovaries and the CNS arise from, and go to, the hypothalamus and extra-hypothalamic areas [3–7]. The sensory nerves innervating the ovary contain various neurotransmitters, such as substance P (SP), vasoactive intestinal peptide (VIP), and calcitonin gene-related peptide (CGRP) [8–11], galanin [12] and neurokinin A and B [13].

Various studies have reported the participation of sympathetic innervation in the regulation of ovarian steroidogenesis [14–19], ovulation [15, 16, 18] and follicular growth [20, 21]. According to Hirshfield [22], the first follicles to start growing are those assembled near the ovarian hilum, which, in the ovaries of the rat, is the first region to be innervated during its feto–neonatal life [23]. According to Lara *et al.* [17], the sympathetic chemical denervation induced by the injection of guanethidine to rats, starting on the 7th day after birth, resulted in an increase in the number of antral follicles and a decrease in the number of preantral follicles. We previously demonstrated that unilateral sectioning of the SON in 16-day-old rats resulted in a significant decrease in the number of ova shed by the denervated ovary and compensatory ovulation by the innervated one [16]. Additionally, we found that the sequential injection of gonadotropins did not restore ovulation by the denervated ovary [18]. Guanethidine-denervation treatment of new-born rats with a pregnant mare’s serum gonadotropin and human chorionic gonadotropin at days 18 or 21 induced ovulation, but this response was not observed when guanethidine was injected into 15-day-old rats [19].

The sensory fibers innervating the ovary are classified as unmyelinated or C-type primary afferent nerves. In the rat, the sensory fibers are permanently destroyed by treatment with the neurotoxin capsaicin [24].

According to Nance *et al.* [25], intrathecal capsaicin treatment had no effect on the estrous cycle, the compensatory ovary hypertrophy (COH), or female sexual behavior. In previous studies, we showed that the injection of capsaicin to adult rats resulted in higher ovarian noradrenaline levels than in the control ones [26].

The subcutaneous injection of capsaicin to newborn female rats resulted in lower monoaminergic activity in the hypothalamus when rats reached 20 days of life [27]. Moreover, we have previously shown that in rats, treatment with capsaicin on the third day of life resulted in a decrease in serum estradiol and progesterone concentrations and that this result was not observed when the treatment with capsaicin was performed in new-born rats [28]. We have demonstrated that in the adult rat, ovarian sensorial innervation participates in the regulation of estradiol secretion in an inhibitory way and that the ovarian sensorial innervation is a neural component. This neural component participates in the establishment of the neuroendocrine conditions regulating the ovarian functions, and its participation varies throughout the estrous cycle [26].

According to Luthman *et al.* [29], an extensive sensory denervation with capsaicin during development can induce an increase in noradrenaline levels in the sympathetic nerve terminals in a target area (rat iris) with a rich SP-ergic sensory innervation, although the sympathetic terminal density is not influenced [29].

Terenghi *et al.* [30] showed that the effects of surgical sympathectomy and sensory denervation induced by capsaicin injection on the morphological relationship between the sensory and the sympathetic nerves in the eye and the oral cavity are different. Surgical sympathectomy resulted in an increase in the number of CGRP-containing fibers, while tyrosine hydroxylase (TH) immunoreactive fibers were totally depleted. On the other hand, capsaicin treatment resulted in an increase of TH-immunoreactive nerves associated with a decrease of CGRP-immunoreactive nerves. These results suggest that there is a close interaction between the sensory and the sympathetic nervous systems, which depends on the developmental stages of the animal [30].

The aim of the present study was to test the hypothesis that there is a synergistic participation of the sensorial and sympathetic neuronal signals in modulating the reactivity of the ovarian compartments to neuroendocrine signals resulting in puberty, onset of ovulation and hormone secretion in the rat. For these purposes, SON sectioning in infantile or juvenile rats with sensorial denervation induced by capsaicin injection to new-born rats was analyzed for effects on puberty, ovulation, serum progesterone, and estradiol concentrations.

## Methods

### Ethics of experimentation

All experiments were carried out in strict accordance with the Mexican Law of Animal Treatment and Protection Guidelines and the specifications in the Mexican Official Standard, NOM-062-ZOO-1999. The Committee of the Facultad de Estudios Superiores Zaragoza approved the experimental protocols. All efforts were made to minimize the number of animals used and their suffering.

Capsaicin (M-2028-Sigma chemical Co., St. Mo. USA) was dissolved in 10 % ethanol, 10 % Tween-80, and 80 % saline (vehicle). The vehicle or capsaicin solutions were injected subcutaneously.

### Animals

Newborn CIIZ-V strain female rats were used. All animals were housed in an artificial light–dark cycle (lights on 05.00 to 19.00 h) with food and water *ad libitum.* On the day of birth (day 0), the new-born offspring were sexed and then were placed in litters of 6 individuals (five females and one male per cage) to avoid disruptions in the developing central nervous system and the reproductive tract of the animal. The new-born rats were injected once with 50 mg/bw of capsaicin in 100 μl per gram of body weight.

The offspring remained with their mother until they were weaned at 21 days of age. At 20 or 28 days of life, animals injected with capsaicin or vehicle were allotted randomly to one of the following experimental groups:

Groups of 8–10 rats injected at birth with capsaicin or vehicle were submitted to a sham operation.

Groups of 8–10 rats injected at birth with capsaicin or vehicle were submitted to uni-or bilateral sectioning of the SON.

### Surgical procedure

#### Sham operation

Sham operation procedures were performed between 10:00 and 12:00 h. Animals were anesthetized with ether, and an incision was performed, including skin and muscle; immediately after, the wound was sutured.

### Sectioning of the superior ovarian nerve (SSON)

Sectioning of the SON was performed between 10:00 and 12:00 h, following previously described methodology [15, 16, 18]. Briefly, animals were anesthetized with ether, a unilateral or bilateral dorsolateral incision was performed, including skin and muscle, and one or both ovaries were exposed. With the aid of fine forceps, the ovarian ligament was sectioned at approximately 1 cm from the ovary. The ovary was subsequently returned to the abdominal cavity, and the wound was sealed.

After surgery animals were returned to their cages. The age of vaginal opening was recorded, and daily vaginal smears were taken thereafter.

### Autopsy procedure

The animals were killed by decapitation between 10:00 and 12:00 h on the day of puberty (first vaginal estrus). The blood of the trunk was collected, allowed to clot and centrifuged at 3000 rpm. The serum was stored at–20 °C until steroid hormones were measured by specific radioimmunoassay. At autopsy, the oviducts were dissected and the number of ova shed was counted, with the aid of a dissecting microscope.

### Hormone measurement

Serum concentration of progesterone (ng/ml) and estradiol (pg/ml) were measured using radioimmunoassay, with kits purchased from Diagnostic Products (Los Angeles, CA, USA). The intra-assay coefficients of variation were 8.35 % and 8.12 % for progesterone and estradiol, respectively, while the inter-assay coefficients of variation were 9.45 % and 9.28 % for progesterone and estradiol, respectively.

### Statistical analyses

Data on the progesterone and the estradiol levels were analyzed using analysis of variance (ANOVA), followed by Tukey’s test. When two means were compared, we used a Student’s *t*-test. The age of first vaginal estrus and the number of ova shed by ovulating animals were analyzed using a Kruskal-Wallis test, followed by a Mann–Whitney *U*-test. The ovulation rate (number of ovulating animals/number of treated animals) was analyzed using a Chi square test. A *p*-value of less than 0.05 was considered significant [26].

## Results

### Age of puberty

The age of puberty of rats injected with capsaicin or vehicle and submitted to a sham operation on day 20 of life was similar. In rats injected with capsaicin, the unilateral (left) or bilateral SSON delayed puberty (Table 1).

**Table 1:** Age of vaginal opening (days)

Pharmacological procedure in new born and surgical procedure in 20-day-old rats		
	Vehicle	Capsaicin
SO	38.4 ± 0.8	38.3 ± 0.9
SSON-L	37.3 ± 0.4	40.4 ± 0.8^a^
SSON-R	37.5 ± 0.6	38.9 ± 1.0
SSON-B	36.9 ± 0.8	40.8 ± 1.0^b^
Pharmacological procedure in new born and surgical procedure in 28-day-old rats		
	Vehicle	Capsaicin
SO	37.4 ± 0.8	40.6 ± 0.9^c^
SSON-L	37.3 ± 0.9	38.0 ± 0.7
SSON-R	37.7 ± 0.7	39.6 ± 1.2
SSON-B	39.9 ± 0.7	38.3 ± 0.6

Mean ± SEM of the age vaginal opening in animals treated with vehicle or capsaicin at birth and subject at section of the SON. At 20 or 28 days of age, the animals were subjected to a sham-operation (SO) or to left (L), right (R) or bilateral (B) SSON. All animals were sacrificed at the first vaginal estrus. Significance was noted as indicated. ^a^ *p* <0.05 CAPSAICIN + SSON-L vs. VEHICLE + SSON-L at 20 days old. ^b^ *p* <0.05 CAPSAICIN + SSON-B vs. VEHICLE + SSON-B at 20 days of age. ^c^ *p* <0.05 CAP + SO vs. VEHICLE + SO at 28 days of age; Kruskal-Wallis test followed by the Mann–Whitney test

The sham operation performed at 28 days of life in rats injected with capsaicin delayed the age of puberty when compared with the vehicle group. The uni-or bilateral SSON did not modify puberty in either the vehicle or the capsaicin-injected rats (Table 1).

### Ovulation rate

In rats injected with vehicle or capsaicin and submitted to a sham operation at day 20 or 28 of life, the ovulation rates by the left and the right ovary were similar (Table 2).

**Table 2:** Ovulation rate by ovary (%)

Pharmacological procedure in new born and surgical procedure in 20-day-old rats								
	*Vehicle*	*Capsaicin*	*Vehicle*	*Capsaicin*	*Vehicle*	*Capsaicin*	*Vehicle*	*Capsaicin*
	*SO*	*SO*	*SSON-L*	*SSON-L*	*SSON-R*	*SSON-R*	*SSON-B*	*SSON-B*
LEFT OVARY	89	100	21^ab^	55	67	71	60	75
RIGHT OVARY	100	100	93	91	33^ac^	71	70	75
Pharmacological procedure in new born and surgical procedure in 28-day-old rats								
	*Vehicle*	*Capsaicin*	*Vehicle*	*Capsaicin*	*Vehicle*	*Capsaicin*	*Vehicle*	*Capsaicin*
	*SO*	*SO*	*SSON-L*	*SSON-L*	*SSON-R*	*SSON-R*	*SSON-B*	*SSON-B*
LEFT OVARY	73	100	50^ab^	89	100	89	75	83
RIGHT OVARY	91	100	100	100	70	56	75	100

Percent of ovulation rate in animals treated with vehicle or capsaicin at birth and subjected to SON sectioning. Animals were treated with vehicle or capsaicin at birth. At 20 or 28 days of age, the animals were subjected to a sham-operation (SO) or were subjected to left (L), right (R) or bilateral (B) SSON. All animals were sacrificed at the first vaginal estrus. Significance was noted as indicated. ^a^ *p* <0.05 VEHICLE + SSON-L or VEHICLE + SSON-R vs. the contralateral ovary of the same group, Chi square test. ^b^ *p* <0.05 VEHICLE + SSON-L (left ovary) vs. VEHICLE + SO (left ovary), Chi square test. ^c^ *p* <0.05 VEHICLE + SSON-R (right ovary) vs. VEHICLE + SO (right ovary), Chi square test

On the other hand, the unilateral SSON at 20 days of age in groups of animals treated with vehicle at birth resulted in a decreased ovulation rate due to the sympathetically denervated ovary (Table 2). In animals injected with capsaicin and submitted to unilateral SSON at 20 days of age, this response was not observed, and the sensorially denervated ovary maintained its ovulatory capacity (Table 2).

The left SSON at 28 days of age in groups of animals treated with vehicle at birth resulted in a decreased ovulation rate by the sympathetically denervated ovary, while the ovulation rate by the ovary with its sympathetic innervation intact was not modified (Table 2). Right SSON at 28 days of age to vehicle-or capsaicin-injected animals did not modify the spontaneous ovulation rate (Table 2).

Bilateral SSON at 20 or 28 days of age to capsaicin-injected animals did not modify the spontaneous ovulation rate as compared with the groups injected with vehicle (Table 2).

### Number of ova shed

#### Effects of sham operation

In rats treated with vehicle, sham surgery on 20-day-old rats resulted in a higher number of ova shed by the right ovary than by the left one. The number of ova shed by the right ovary of capsaicin-treated rats was lower than that shed by the vehicle-treated group. Such differences were not observed in rats treated at 28 days of life (Table 3).

**Table 3:** Number of ova shed by ovary

Pharmacological procedure in new born and surgical procedure in 20-day-old rats								
	*Vehicle*	*Capsaicin*	*Vehicle*	*Capsaicin*	*Vehicle*	*Capsaicin*	*Vehicle*	*Capsaicin*
	*SO*	*SO*	*SSON-L*	*SSON-L*	*SSON-R*	*SSON-R*	*SSON-B*	*SSON-B*
LEFT OVARY	3.8 ± 0.7	5.3 ± 0.9	0.9 ± 0.6^ab^	3.2 ± 1.0	7.2 ± 0.6^b^	4.1 ± 1.1^e^	4.8 ± 1.4	3.8 ± 1.0
RIGHT OVARY	5.8 ± 0.6^a^	3.7 ± 0.5^c^	6.5 ± 0.6	5.6 ± 0.9	2.5 ± 1.2^ac^	2.7 ± 0.8^c^	5.0 ± 1.4	2.5 ± 0.4^c^
Pharmacological procedure in new born and surgical procedure in 28-day-old rats								
	*Vehicle*	*Capsaicin*	*Vehicle*	*Capsaicin*	*Vehicle*	*Capsaicin*	*Vehicle*	*Capsaicin*
	*SO*	*SO*	*SSON-L*	*SSON-L*	*SSON-R*	*SSON-R*	*SSON-B*	*SSON-B*
LEFT OVARY	3.1 ± 0.7	4.9 ± 1.1	2.3 ± 0.8^a^	4.2 ± 0.7	5.2 ± 0.5	5.0 ± 1.1	3.0 ± 3.1	2.3 ± 0.7
RIGHT OVARY	4.4 ± 0.8	4.5 ± 0.9	6.2 ± 0.8	4.8 ± 0.5	2.8 ± 0.7^a^	2.0 ± 0.6 ^acd^	3.6. ± 1.1	3.5 ± 0.9

Mean ± SEM number of the ova shed in animals injected at birth with vehicle or capsaicin and subjected to SON sectioning. The rats were injected with vehicle or capsaicin at birth. At 20 or 28 days of age, the animals were subjected to a sham-operation (SO) or to left (L), right (R) or bilateral (B) SSON. All animals were sacrificed at the first vaginal estrus. Significance was noted as indicated. ^a^ *p* <0.05 VEHICLE + SO, VEHICLE + SSON-L or VEHICLE + SSON-R vs. the contralateral ovary of same group. ^b^ *p* <0.05 VEHICLE + SSON-L (left ovary) vs. VEHICLE + SO (left ovary). ^c^ *p* <0.05 VEHICLE + SSSON-R (right ovary) vs. VEHICLE + SO (right ovary). ^d^ *p* <0.05 CAPSAICIN + SSON-R (right ovary) vs. CAPSAICIN + SO (right ovary). ^e^ *p* <0.05 CAPSAICIN + SSON-R (left ovary) vs. VEHICLE + SSON-R (left ovary). Kruskal-Wallis test followed by the Mann–Whitney test

#### Effects of unilateral or bilateral SSON

The number of ova shed by the left ovary (sympathetic denervated ovary) when left SSON was performed on 20-or 28-days old vehicle-treated rats was lower than the ova shed by the right one; and lower than the ova shed by the left ovary of animals who underwent sham operations. These effects were not observed in rats injected with capsaicin and submitted to the same procedure (Table 3).

Right SSON on 20-day-old vehicle-treated rats resulted in a diminished number of ova shed by the right ovary when compared with the contralateral ovary or the right ovary of the vehicle-treated and sham operation group (Table 3).

Right SSON on 20-day-old animals injected with capsaicin resulted in a decreased number of ova shed by the left ovary (ovary with sympathetic innervation intact) when compared with the left ovary of the group treated with vehicle and subjected to right SSON (Table 3).

Right SSON on 28-day-old animals treated with vehicle resulted in a diminished number of ova shed by the right ovary when compared with the contralateral ovary. Right SSON on animals injected with capsaicin resulted in a decreased number of ova released by the right ovary (sympathetic denervation ovary) as compared to the contralateral ovary or the right ovary of the capsaicin and sham operation group (Table 3).

The bilateral sectioning of the SON at 20 or 28 days of age to capsaicin-injected animals did not modify the number of ova shed when compared with the group treated with vehicle and submitted to the same treatment (Table 3).

### Progesterone serum levels (Fig. 1)

**Fig. 1:**
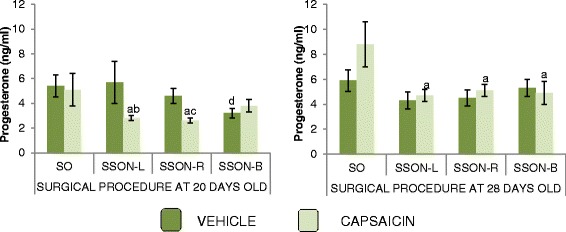
Progesterone serum levels. Mean ± SEM of progesterone serum levels (ng/ml) in animals injected at birth with vehicle or capsaicin and subjected to SON sectioning. At 20 or 28 days of age, the animals were subjected to a sham-operation (SO) or left (L), right (R) or bilateral (B) SSON and then sacrificed at the first vaginal estrus. Significance was noted as indicated. ^a^ *p* <0.05 VEHICLE + SO vs. CAPSAICIN + SSON-L; −R at 20 days old, and VEHICLE + SO vs. CAPSAICIN + SSON-L; −R or -B, at 28 days of age, ANOVA test followed Tukey’s test. ^b^ *p* <0.05 VEHICLE + SSON-L vs. CAPSAICIN + SSON-L, at 20 days old, Student’s *t* test. ^c^ *p* <0.05 VEHICLE + SSON-R vs. CAPSAICIN + SSON-R, at 20 days old, Student’s *t* test. ^d^ *p* <0.05 VEHICLE + SO vs. VEHICLE + SSON-B, at 20 days of age, ANOVA test followed Tukey’s test

In rats injected with vehicle or capsaicin and submitted to the sham operation at the age of 20 or 28 days, the progesterone levels were similar.

In comparison with the sham operation group, unilateral SSON performed at 20 or 28 days of life to rats injected with the vehicle did not modify the progesterone levels. Bilateral SSON performed at 20 days of life resulted in lower progesterone levels.

Unilateral SSON in capsaicin-injected rats, when performed at the age of 20 days, resulted in lower progesterone levels than in vehicle-injected rats submitted to the same surgery or capsaicin-injected rats submitted to sham operation.

When uni-or bilateral SSON was performed at day 28 of life on capsaicin-injected rats, the progesterone levels were lower than in capsaicin-injected rats submitted to sham operation.

### Estradiol serum levels (Fig. 2)

**Fig. 2:**
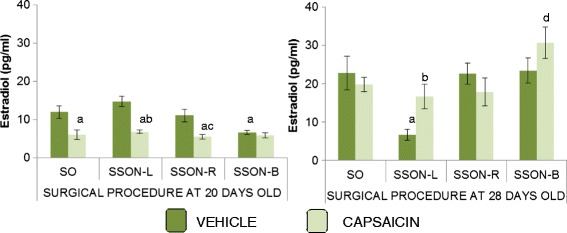
Estradiol serum levels. Mean ± SEM of estradiol serum levels (pg/ml) in animals injected at birth with vehicle or capsaicin and subjected to SON sectioning. At 20 or 28 days of age, the animals were subjected to a sham-operation (SO) or to left (L), right (R) or bilateral (B) SSON and sacrificed at the first vaginal estrus. Significance was noted as indicated. ^a^ *p* <0.05 VEHICLE + SO vs. CAPSAICIN + SO, CAPSAICIN + SSON-L or R and VEHICLE + SSON-B at 20 days old, and VEHICLE + SO vs. VEHICLE + SSON-L at 28 days of age, ANOVA test followed Tukey’s. ^b^ *p* <0.05 vehicle + SSON-L vs. CAPSAICIN + SSON-L at 20 or 28 days of age, ANOVA test followed Tukey’s. ^c^ *p* <0.05 VEHICLE + SSON-R vs. CAPSAICIN + SSON-R at 20 days old, Student’s *t* test. ^d^ *p* <0.05 CAPSAICIN + SO vs. CAPSAICIN + SSON-B at 28 days old, Student’s *t* test

Estradiol levels were lower in capsaicin-injected rats submitted to sham operation at the age of 20 days. These differences were not observed in rats with sham operation performed at 28 days of life.

Bilateral SSON on 20-day-old rats injected with vehicle decreased estradiol levels when compared with the sham operation group.

Unilateral SSON to capsaicin-injected rats, performed at the age of 20 days, showed lower estradiol levels than in rats injected with vehicle and submitted to the same surgery or a sham operation.

The estradiol levels in vehicle-injected rats with left SSON performed at day 28 of life were lower than in the sham operation group. In capsaicin-injected rats subjected to left SSON, the estradiol serum levels were higher than in the vehicle-injected rats with the same surgery.

In comparison with the sham operation, unilateral SSON on rats injected with capsaicin did not modify estradiol levels. Bilateral SSON on capsaicin-injected rats resulted in higher estradiol levels than in rats submitted to a sham operation.

## Discussion

The results obtained in the current study suggest that besides its regulation by the hormones of the hypothalamic-pituitary-ovarian axis, the onset of puberty is also regulated by the sensory information carried by the vagus nerve and the sympathetic information reaching the ovary through the SON.

Delayed puberty resulted from the peripheral pharmacological sympathetic denervation by guanethidine injection to seven-day-old rats [17], sensory denervation performed by capsaicin injection to three-day-old rats [28] and bilateral SSON to 4-day-old rats [31]. Unilateral or bilateral SSON performed at 24 [2] or 16 [16] days of life did not affect the onset of puberty, similar to the present study, suggesting that the participation of sympathetic ovarian innervations in the onset of puberty, an estrogen-dependent event, varies with the development stage of the animal.

Another possibility is that pharmacological sympathetic or sensory denervation affected the hormonal secretion by the adrenal glands because medullectomy (removal of most of the adrenal medulla without compromising adrenocortical function) delays puberty [32].

In the present study, the sensory denervation at birth followed by left or bilateral SSON performed at 20 days of life also delayed puberty, suggesting a bidirectional regulation between the sympathetic and sensory fibers.

The destruction of ovarian nerve fibers in neonatal rats by treatment with antibodies to nerve growth factor (ab-NGF) resulted in failure of development of the sympathetic (noradrenergic and neuropeptide-Y) nerves. Partial loss of sensory innervation, represented by the calcitonin gene-related peptide fibers, was also observed. The timing of first ovulation was delayed, the estrous cycle was disrupted, and fertility was compromised [33]. Because the ab-NGF injection affected the development of many kinds of fibers, it should be noted that in the absence of NGF, not only sympathetic fibers die but also a group of sensory fibers die, particularly the type C, capsaicin-sensitive fibers [34]. These data suggest that the neuroendocrine mechanisms leading to puberty include the hormone environment along with sympathetic and sensory information.

Gerendai and Halaz [35] described the existence of several asymmetries in the anatomy of the peripheral innervations. According to Klein and Burden [36], the right ovary receives more sympathetic afferent fibers than the left one, while Tóth et al. presented evidence that the CNS representation of the left ovary is higher than the right one [7]. In present study, we observed that the effects of unilateral SSON depend on the nerve sectioned, suggesting that each ovary is regulated in a different way by the information arriving by the SON.

Unilateral ovarian sympathectomy by local administration of 6-hydroxy dopamine in one of the ovaries results in an increase in the number of ova shed by the innervated ovary, similar to the observations in a hemi-spayed animal [37]. The present results show an increase in the ova shed in rats with unilateral SSON that depends on sensory innervation integrity, suggesting that sensory innervation modulates the ovarian sympathetic innervation, as Simmons proposed [38].

Finally, we cannot exclude that sensory innervation has a direct effect on the events that culminate in ovulation. Calka et al. [39] observed immunoreactive CGRP fibers alongside the developing follicles, so that removal of sensory fibers could significantly modify the microenvironment around the follicle and, thus, the final maturation of the follicle that culminates in ovulation.

Previously, we showed that ovarian and systemic injection of capsaicin in adult rats resulted in higher estradiol, progesterone serum levels and ovarian noradrenaline [26, 40]. This increase could be the result of an increase in the intra-ovarian noradrenaline concentration of the animals injected with capsaicin. In the adult rat, bilateral sectioning of the ovarian superior nerve resulted in a decrease in the estradiol and intra-ovarian noradrenaline concentrations, and it has been proposed that noradrenergic innervation stimulates estradiol secretion [41, 42].

We previously proposed that the sensory ovarian innervation participates in regulating steroidogenesis [26]. The results obtained in the present study suggest that the participation of such innervation in the regulation of estradiol secretion is more prominent in the infantile rat (day 20) than in the juvenile rat (day 28).

## Conclusions

Studies by Stener-Victorin et al. [43] and Lara et al. [44] suggest that the polycystic ovary syndrome should include the participation of the ovarian innervations in the physiopathology of the syndrome. Present and other experimental studies support such idea [43, 44]. Based on the present results, we suggest that sympathetic innervation regulates ovulation and the secretion of steroid hormones and that the sensory fibers modulate the sympathetic innervation action on ovarian functions.

## Abbreviations

CGRP: Calcitonin gene-related peptide
CNS: Central nervous system
COH: Compensatory ovary hypertrophy
FSH: Follicle-stimulating hormone
LH: Luteinizing hormone
NGF: Nerve growth factor
SO: Sham-operation
SON: Superior ovarian nerve
SP: Substance P
SSON: Sectioning of the superior ovarian nerve
TH: Tyrosine hydroxylase
VIP: Vasoactive intestinal peptide
